# Factors Associated With Early Elementary Child Health-Related Quality of Life: The Generation R Study

**DOI:** 10.3389/fpubh.2021.785054

**Published:** 2022-01-27

**Authors:** Yueyue You, Amy van Grieken, Fernando Estévez-López, Junwen Yang-Huang, Hein Raat

**Affiliations:** ^1^The Generation R Study Group, Erasmus Medical Center, Rotterdam, Netherlands; ^2^Department of Public Health, Erasmus Medical Center, Rotterdam, Netherlands; ^3^Department of Child and Adolescent Psychiatry/Psychology, Erasmus Medical Center, Rotterdam, Netherlands

**Keywords:** socio-demographic characteristics, socioeconomic status, health-related lifestyle behaviors, chronic conditions, HRQOL, early elementary school age children

## Abstract

**Aim:**

To identify the factors associated with health-related quality of life (HRQOL) among early elementary age children (5–6 years) from a general population sample.

**Methods:**

We analyzed data of 4,202 children from the Generation R Study, a population-based cohort study in the Netherlands. Children's physical and psychosocial HRQOL were measured using the Child Health Questionnaire Parent Form 28 (CHQ-PF28). Associations between socio-demographic characteristics (child age, sex, ethnic background, family situation, parental educational level, parental employment status, and net household income), health-related lifestyle behaviors (physical activity and screen time), health conditions (number of chronic conditions, emotional and behavioral problems, and family functioning) and children's physical and psychosocial HRQOL were assessed using multivariate regression analyses.

**Results:**

Mean child age was 6.0 years (SD: 0.43); 63.6% had a majority (Dutch) ethnic background. Children with a non-western ethnic background, and children of unemployed mothers had a lower physical HRQOL (all *p* < 0.05). Older children, boys, and children from single-parent or low educated families had a lower psychosocial HRQOL (all *p* < 0.05). Children from a low income household family, children having chronic conditions or emotional and behavioral problems, or from families with relatively high “pathological family functioning” reported both lower physical and psychosocial HRQOL (all *p* < 0.05).

**Conclusion:**

Indicators of adverse socioeconomic and family circumstances and indicators of child health problems were associated with lower HRQOL. Public health initiatives to improve HRQOL of children should prioritize children from a low socioeconomic status or with less favorable health conditions from early age onwards.

## Introduction

Health-related quality of life (HRQOL) is a broad, multidimensional construct including not only disease states but also the person's physical, psychological, and social well-being (1). It is been increasingly used as an additional health outcome among children to assess their physical and social functioning, physical and mental health, and to evaluate the effect of both clinical and population-based interventions (2). Identifying the factors that affect child HRQOL may help develop effective prevention programs for children, and thereby promote positive physical and psychosocial health in the long term (3).

Several models have been proposed to study factors associated with HRQOL (4–6). For example, a general model from Wilson and Cleary (7) suggests five levels of factors related to HRQOL: biological, physiological, symptoms, functioning, and general health. Other researchers evaluating potential determinants of HRQOL have suggested that certain factors might have differential associations with HRQOL than other factors. For example, Sprangers et al. (4) proposed that socio-demographic characteristics (e.g., age, education, household income, etc.) are important factors that can affect individuals' HRQOL. Wendel-Vos et al. (5) suggested that health-related lifestyle behavior (e.g., physical activity, smoking, etc.) is an important predictor of HRQOL in both children and adults. In addition, Ware et al. (6) suggested that health conditions (e.g., overweight/obesity, asthma, infectious disease, etc.) have the most immediate direct impact on HRQOL. Thus, identifying whether and how the factors from these multiple domains have an impact on children's HRQOL is important to subgroups of children who are at risk of impaired HRQOL.

However, in the literature so far, the findings about associations between socio-demographic characteristics and HRQOL are not consistent (8, 9). For example, a study among U.S. school-aged children found that parental educational level was not related to child HRQOL, but household income was positively related to child overall HRQOL (10). Another study among European school-aged children reported that maternal educational level and household income were both positively related to child psychosocial HRQOL (11). Furthermore, previous studies mainly focused on one domain; research that studies simultaneously the associations of factors covering three domains (socio-demographic characteristics, health-related lifestyle behaviors, and health conditions) and HRQOL are lacking among young children. A comprehensive understanding of the potential factors associated with HRQOL provides support for intervention development and public health practitioners focusing on improving child HRQOL. Thus, in this study, we selected multiple factors covering the three domains relevant for HRQOL: socio-demographic characteristics, health-related lifestyle behaviors of the child, and indicators of health and well-being of the child and family.

The aim of the present study was to identify which factors were associated with both physical and psychosocial HRQOL at early elementary school age (5–6 years old). All factors were studied across three domains: socio-demographic characteristics (child age, sex, ethnic background, parental educational level, parental employment status, and net household income), health-related lifestyle behaviors (physical activity and screen time of the child), and indicators of health and well-being of the child and family (number of chronic conditions, emotional and behavioral problems, and family functioning). Based on previous studies (12–14), the hypothesis was that children with higher socio-demographic status; children with more favorable health-related behaviors; and children with more favorable health, well-being, and family functioning are more likely to have higher HRQOL than their counterparts.

## Methods

### Study Design and Population

This cross-sectional study was embedded in the Generation R Study, a multi-ethnic prospective cohort study of children born between 2002 and 2006 in the city of Rotterdam, the Netherlands, that has been described previously (15). In total, 8,305 children aged 6-years old still participated in the ongoing cohort study, and relevant data were collected from 2008 to 2012. Among participants of 8,305, parents of 5,298 joined the measurement of their child's HRQOL; parents did not finish the full questionnaire of measurement of HRQOL (*n* = 563) were excluded. To avoid clustering of data, second (*n* = 505) and third children (*n* = 12) from the same family were excluded. Then, children with missing data on all determinants (*n* = 16) were also excluded. Leaving a study population of 4,202 participants. The study was approved by the Medical Ethics Committee of the Erasmus University Medical Centre (MEC 217.595/2002/202). Written informed consent was obtained from all participants.

### Independent Variables

Socio-demographic characteristics, health-related lifestyle behaviors, and indicators of health and well-being of the child and family were considered as the factors of HRQOL in this study. All related information was obtained via parental questionnaire when the child was 5 to 6 years old.

Socio-demographic characteristics included child age (years), sex (boy, girl), ethnic background (Dutch, other western, non-western), family situation (two-parent family, one-parent family), parental educational level (high, middle, low), parental employment status (paid job, no paid job), and net household income (>€3,200, €2,000–3,200, < €2,000/month). Child ethnic background was based on the country of birth of the parents [according to the classification of Statistics Netherlands (16)]. Children were categorized into three groups regarding ethnic background: Dutch, other Western (American western, Asian western, European and Oceania), and non-Western (Indonesian, Cape Verdean, Moroccan, Dutch Antilles, Surinamese, Turkish, African, American non-western and Asian non-western). Both mother and father educational levels were defined according to Statistics Netherlands and categorized into three educational levels: high (bachelor's degree, higher vocational school or a university degree finished), middle (>4 years general secondary school, or intermediate vocational school), and low (no education, primary education, ≤ 4 years general secondary school, or lower vocational training) (17).

Indicators of health-related lifestyle behaviors included physical activity and screen time at child age 6 years. Child physical activity refers to sports participation and outdoor play. For sports participation, parents were asked the following question: “Does your child take part in sports (e.g., football, judo, tennis, etc.?).” Then, frequency (number of days) and duration (minutes) were asked for weekdays and weekend days separately. The average sports participation time per day of the child was calculated by the following formula: [(days per week) ^*^ (hours on a weekday) + (days per weekend) ^*^ (hours on a weekend day)]/7. The same set of questions was used to assess children's time spent on outdoor playing. Then, physical activity time per day was calculated by adding up children's sports participation and outdoor play. According to new WHO guidelines on physical activity for children, physical activity time was dichotomized into <1 hour per day and ≥1 hour per day (18). Child screen time refers to television viewing (including video/DVD) and computer game use (including video games). For both variables, frequency (number of days) and duration (minutes) were asked for weekdays and weekend days separately. Then, screen time per day was calculated by adding up children's television viewing and computer game use, and was dichotomized at <2 h per day and ≥2 h per day according to the recommendation from the American Academy of Pediatrics (19).

With regard to indicators of health and well-being of the child and family, we included the number of chronic conditions, the presence of emotional and behavioral problems, and the presence of “pathological family functioning.” Information of the chronic condition were measured by parents-reported questionnaire when the child aged 6-years old. Parents were asked whether their child had infectious disease (ear infection, urinary tract infection or bladder infection, sore throat, spurious croup, bronchitis, pneumonia, impetigo, stomach flu and/or diarrhea), wheezing attack, eczema, and pain in the past 12 months. Number of chronic conditions was counted based on parent's reply, and then categorized as 0, 1, 2, and 3 or more chronic conditions. Presence of emotional and behavioral problems (yes/no) were assessed with the Dutch version of Child Behavior Checklist (CBCL/1.5-5), a 99-item parents-reported questionnaire (20). The CBCL/1.5-5 has shown good concurrent validity and test-retest reliability in Dutch children (21). A borderline cut-off score [83^rd^ percentile of a Dutch norm group (22)] of the CBCL total problem score was used to differentiate between children with and without emotional and behavioral problems (23). Family functioning (pathological/not pathological) was measured with the 12-item General Function scale of the McMasters Family Assessment Device (FAD) (24), a score >2.17 was defined as “pathological,” a score ≤ 2.17 was defined as “not pathological” (25). The McMaster FAD is a validated measure assessing overall health and pathology of a family (25).

### Health-Related Quality of Life

With regard to HRQOL of their child, parents completed the Child Health Questionnaire Parent Form 28 items (CHQ-PF28), of which the validity and reliability had been documented (26). The questionnaire consists of 28 items with four, five or six response options across eight multi-item scales and five single-item concepts. The items from each of the scales were summed and transformed into 0 to 100 (0 indicating worst health and 100 best health). According to the CHQ Manual, these scores are used to calculate the Physical Summary Component Scale (PhS) and the Psychosocial Summary Component Scale (PsS); summary scores of 50 represent the mean in the US reference population children; 10 points above/below 50 reflect one standard deviation difference in either direction (26, 27).

### Data Analyses

Descriptive analysis was applied to characterize the study population. Physical and psychosocial HRQOL across different factors were assessed using independent sample *t*-tests and one-way ANOVA (see Appendix Table 1). The normality of the data (the summary scores) was examined. Although score distributions were slightly negatively skewed, with the large number of respondents, parametric tests were performed (28). The multi-collinearity among all potential factors influencing HRQOL was assessed by examining the variance inflation factor (VIF) values. All the VIF values were <3, indicating acceptable collinearity between all indicators. Multivariate linear regressions were applied to assess the factors of physical and psychosocial HRQOL. In model 1, all socio-demographic characteristics were entered to model. In model 2, with additional inputs all indicators of health-related behaviors. In model 3, with additional inputs all indicators of health and well-being of the child and family. In Appendix Table 1, we calculated effect sizes (Cohen's d) to assess the relative differences between specific subgroups with regard to the PhS and PsS scores. The effect sizes (d) were calculated by dividing the difference in mean scores between subgroups by the largest SD (small difference: 0.2 ≤ d <0.5; moderate difference: 0.5 ≤ d <0.8; large difference: d ≥ 0.8) (29). Multiple imputation was applied to impute missing data in the determinants (30). Ten imputed datasets were generated using a fully conditional specified model, taking into account all the variables included in this study. Pooled estimates from these ten imputed datasets were used to report beta coefficients and 95% confidence intervals (95% CI). All analyses were conducted with Statistical Package for Social Sciences (SPSS) version 21.0 for Windows (IBM Corp., Armonk, NY, USA). Significance differences were indicated at the level of *p* < 0.05.

### Non-response Analyses

Children with missing data on HRQOL (*n* = 563) were compared with children without missing data (*n* = 4,202) using Chi-square tests. Data were more often missing for children whose parents had a low educational level, parents without a paid job, a low household income, a single-parent family, from non-western ethnic background, and with screen time more than 2 h per day (all *p* < 0.05).

## Results

Table 1 summarizes the general characteristics of the study population. Mean child age was 6.0 years (SD: 0.43); 2,090 (49.7%) were girls; 63.6% had a majority (Dutch) ethnic background. The average score (SD) for physical and psychosocial summary score were 56.98 (6.57) and 52.73 (7.07), respectively. The univariate analysis of physical and psychosocial summary score between subgroups according to socio-demographic characteristics, health-related lifestyle behaviors, and health and well-being of the child and family were presented in Appendix Table 1. The results showed that the following factors were associated with a higher physical and psychosocial summary score: two-parent household, high-educated fathers, employed mothers, a high income household, having physical activity more than 1 h per day, screen time <2 h per day, and no chronic disease (all *p* < 0.05).

**Table 1:** General characteristics of the study population among 6-year-old children (*n* = 4,202).

		**Total**	**Missing**
**Socio-demographic characteristics**
Child age (years), mean (SD)		6.02 ± 0.43	144 (3.4)
Child gender, *n* (%)	Girl	2,090 (49.7)	0
	Boy	2,112 (50.3)	
Child ethnic background, *n* (%)	Dutch	2,663 (63.6)	15 (0.4)
	Other western	374 (8.9)	
	Non-western	1,150 (27.5)	
Family situation, *n* (%)	Two-parent family	3,523 (87.6)	179 (4.3)
	One-parent family	500 (12.4)	
Maternal educational level, *n* (%)	Low	456 (11.4)	191 (4.5)
	Mid	1,227 (30.6)	
	High	2,328 (58.0)	
Paternal educational level, *n* (%)	Low	558 (15.0)	485 (11.5)
	Mid	982 (26.4)	
	High	2,177 (58.6)	
Maternal employment status, *n* (%)	Paid job	2,951 (77.2)	380 (9.0)
	No paid job	871 (22.8)	
Paternal employment status, *n* (%)	Paid job	3,424 (94.6)	582 (13.9)
	No paid job	196 (5.4)	
Net household income, *n* (%)	< €2,000/month	785 (20.6)	394 (9.4)
	€2,000–€3,200/month	1,016 (26.7)	
	More than €3,200/month	2,007 (52.7)	
**Health-related lifestyle behaviors**
Physical activity, *n* (%)	<1 hour/day	1,042 (30.8)	822 (19.6)
	≥1 hour/day	2,338 (69.2)	
Screen time, *n* (%)	≥2 hours/day	1,538 (37.9)	144 (3.4)
	<2 hours/day	2,520 (62.1)	
**Health and well-being of the child and family**
Number of chronic conditions, *n* (%)	0	1,011 (28.5)	649 (15.4)
	1	1,385 (39.0)	
	2	799 (22.5)	
	≥3	358 (10.0)	
Emotional and behavioral problems* (Yes), *n* (%)		270 (6.7)	193 (4.6)
Family functioning**, *n* (%)	Not pathological	3,750 (94.5)	232 (5.5)
	Pathological	220 (5.5)	
Child health-related quality of life***
Physical summary score (PhS), mean (SD)		56.98 ± 6.57	0
Psychosocial summary score (PsS), mean (SD)		52.73 ± 7.07	0

The table is based on a non-imputed dataset. *Behavioral problems were assessed with the dutch version of child behavior checklist. **Family functioning was measured with the 12-item general function scale of the mcmasters family assessment device. ***Child health-related quality of life was measured by the child health questionnaire parental form 28.

Table 2 presents the factors associated with physical HRQOL. After including all three domains of factors into the model, non-western ethnic background, maternal unemployment status, and low household income were associated with lower physical HRQOL (all *p* < 0.05). In the domain of health and well-being of the child and family, all factors included were significantly associated with physical HRQOL (all *p* < 0.05). Emotional and behavioral problems, and pathological family functioning were associated with lower physical HRQOL. Having chronic conditions were also associated with lower physical HRQOL, of which having three or more chronic conditions showed the largest reduction in physical HRQOL (β = −4.59; 95%CI: −5.40, −3.77).

**Table 2:** Multivariate analyses of socio-demographic characteristics, health-related lifestyle behaviors, and health and well-being of the child and family with physical summary score (PhS).

	**Independent variables**		
	**Model 1**	**Model 2**	**Model 3**
	**β (95% CI)**	**β (95% CI)**	**β (95% CI)**
***Socio-demographic characteristics***
Age	0.01 (−0.48, 0.49)	0.03 (−0.45, 0.51)	−0.04 (−0.51, 0.43)
**Gender**
Girl	Ref	Ref	Ref
Boy	0.05 (−0.34, 0.44)	0.08 (−0.31, 0.48)	0.10 (−0.28, 0.49)
**Child ethnic background**
Dutch	Ref	Ref	Ref
Other western	−0.12 (−0.83, 0.59)	−0.07 (−0.77, 0.64)	−0.15 (−0.84, 0.54)
Non-western	**−1.04 (−1.54**, −0.53)**	−0.89 (−1.41, 0.38)**	**−0.90 (−1.40**, −0.39)**
**Family situation**
Two-parent family	Ref	Ref	Ref
One-parent family	0.28 (−0.48, 1.03)	0.28 (−0.47, 1.03)	0.46 (−0.31, 1.23)
**Maternal educational level**
High	Ref	Ref	Ref
Middle	0.31 (−0.22, 0.84)	0.35 (−0.19, 0.88)	0.33 (−0.19, 0.86)
Low	0.04 (−0.79, 0.87)	0.11 (−0.73, 0.94)	0.04 (−0.77, 0.85)
**Paternal educational level**
High	Ref	Ref	Ref
Middle	−0.01 (−0.59, 0.57)	0.00 (−0.58, 0.59)	0.05 (−0.52, 0.63)
Low	**-0.77 (−1.51**, −0.02)*	−0.72 (−1.46, 0.02)	−0.63 (−1.37, 0.11)
**Maternal employment status**
Paid job	Ref	Ref	Ref
No paid job	**−0.66 (−1.19**, −0.12)*	**−0.64 (−1.18**, −0.10)*	**−0.60 (−1.16**, −0.04)*
**Paternal employment status**
Paid job	Ref	Ref	Ref
No paid job	−0.03 (−0.99, 0.93)	−0.02 (−0.98, 0.94)	0.02 (−0.97, 1.02)
**Household income**
≥€3,200	Ref	Ref	Ref
€2,000–3,200	−0.55 (−1.11, 0.01)	−0.55 (−1.10, 0.01)	−0.38 (−0.93, 0.18)
< €2,000	**−1.29 (−2.08**, −0.50)**	**−1.24 (−2.03**, −0.45)**	**−1.03 (−1.82**, −0.25)**
***Health-related lifestyle behaviors***
**Physical activity**
<1 hour/day		Ref	Ref
≥1 hour/day		0.39 (−0.12, 0.89)	0.35 (−0.15, 0.85)
**Screen time**	
≥2 hour/day		Ref	Ref
<2 hour/day		0.47 (0.02, 0.91)*	0.38 (−0.06, 0.82)
***Health and wellbeing of the child and family***
**Number of chronic conditions**
0			Ref
1			**−0.92 (−1.66**, −0.19)**
2			**−2.01 (−2.62**, −1.40)**
≥3			**−4.59 (−5.40**, −3.77)**
Emotional and behavioral problems			**−0.84 (−1.65**, −0.03)**
Family functioning			**−2.34 (−3.23**, −1.46)**

The table is based on an imputed dataset. Model 1: Indicators of socio-demographic characteristics were added to the model 1. Model 2: Indicators of socio-demographic characteristics and health-related lifestyle behaviors were added to the model 2. Model 3: Indicators of socio-demographic characteristics, health-related lifestyle behaviors, and health and well-being of the child and family were added to the model 3. *p < 0.05, **p < 0.001, significant p-values in bold.

Table 3 presents the factors associated with psychosocial HRQOL. After including all three domains of factors into the model, child age, being a boy, one-parent family, low maternal education level, and low household income were associated with lower psychosocial HRQOL. All factors from health and well-being of the child and family were significantly associated with psychosocial HRQOL. Emotional and behavioral problems, pathological family functioning, and having three or more chronic conditions were associated with lower psychosocial HRQOL (all *p* < 0.05).

**Table 3:** Multivariate analyses of socio-demographic characteristics, health-related lifestyle behaviors, and health and well-being of the child and family with psychosocial summary score (PhS).

	**Independent variables**		
	**Model 1**	**Model 2**	**Model 3**
	**β (95% CI)**	**β (95% CI)**	**β (95% CI)**
***Socio-demographic characteristics***
Age	**−0.92 (−1.45**, −0.40)**	**−0.89 (−1.42**, −0.36)**	**−0.93 (−1.41**, −0.44)**
**Gender**	
Girl	Ref	Ref	Ref
Boy	**−1.01 (−1.44**, −0.59)**	**−0.97 (−1.40**, −0.55)**	**−0.75 (−1.15**, −0.35)**
**Child ethnic background**
Dutch	Ref	Ref	Ref
Other western	−0.01 (−0.86, 0.84)	0.12 (−0.74, 0.98)	0.24 (−0.56, 1.04)
Non-western	0.39 (−1.15, 0.38)	−0.45 (−1.22, 0.31)	−0.59 (−1.23, 0.12)
**Family situation**
Two-parent family	Ref	Ref	Ref
One-parent family	**−1.38 (−2.22**, −0.53)**	**−1.37 (−2.21**, −0.54)**	**−0.87 (−1.65**, −0.10)*
**Maternal educational level**
High	Ref	Ref	Ref
Middle	**−1.40 (−2.25**, −0.55)**	**−1.48 (−2.34**, −0.63)**	**−1.79 (−2.58**, −1.01)**
Low	**−0.96 (−1.74**, −0.17)**	**−0.99 (−1.78**, −0.20)**	**−1.20 (−1.94**, −0.46)**
**Paternal educational level**
High	Ref	Ref	Ref
Middle	−0.13 (−0.78, 0.53)	−0.11 (−0.77, 0.55)	−0.15 (−0.74, 0.45)
Low	−0.64 (−1.43, 0.16)	−0.58 (−1.38, 0.22)	−0.54 (−1.30, 0.22)
**Maternal employment status**	
Paid job	Ref	Ref	Ref
No paid job	−0.13 (−0.76, 0.50)	−0.11 (−0.74, 0.52)	−0.14 (−0.70, 0.43)
**Paternal employment status**
Paid job	Ref	Ref	Ref
No paid job	0.03 (−0.94, 1.00)	0.04 (−0.93, 1.01)	0.04 (−0.89, 0.97)
**Household income**
≥€3,200	Ref	Ref	Ref
€2,000–3,200	−0.64 (−1.53, 0.25)	−0.57 (−1.47, 0.34)	0.04 (−0.80, 0.89)
< €2,000	**−1.01 (−1.58**, −0.45)**	**−1.01 (−1.58**, −0.44)**	**−0.72 (−1.25**, −0.18)**
***Health-related lifestyle behaviors***
**Physical activity**
<1 hour/day		Ref	Ref
≥1 hour/day		0.62 (0.11, 1.13)*	0.40 (−0.07, 0.86)
**Screen time**
≥2 hour/day		Ref	Ref
<2 hour/day		0.58 (0.10, 1.01)*	0.28 (−0.17, 0.73)
***Health and well-being of the child and family***
**Number of chronic conditions**
0			Ref
1			**−0.68 (−1.23**, −0.13)**
2			**−1.16 (−1.77**, −0.55)**
≥3			**−1.82 (−2.70**, −0.94)**
Emotional and behavioral problems			**−9.37 (−10.25**, −8.50)**
Family functioning			**−2.59 (−3.51**, −1.66)**

The table is based on an imputed dataset. Model 1: Indicators of socio-demographic characteristics were added to the model 1. Model 2: Indicators of socio-demographic characteristics and health-related lifestyle behaviors were added to the model 2. Model 3: Indicators of socio-demographic characteristics, health-related lifestyle behaviors, and health and well-being of the child and family were added to the model 3. *p < 0.05, **p < 0.001, significant p-values in bold.

## Discussion

The present study was designed to assess which factors were related with physical and psychosocial HRQOL among early elementary school age children (5–6 years). The factors covered three domains: socio-demographic characteristics, health-related lifestyle behaviors, and health and well-being of the child and family. Several socio-demographic characteristics (age, sex, ethnic background, family situation, paternal educational level, maternal employment status, and household income) were associated with lower physical or psychosocial HRQOL; indicators of health and well-being of the child and family (number of chronic conditions, emotional and behavioral problem, and family functioning) were associated with lower physical and psychosocial HRQOL. No associations were observed between health-related lifestyle behaviors and HRQOL.

Our findings showed that children with a low family socioeconomic status, as indicated by low maternal education level, maternal unemployment, and low income household have a relatively lower physical or psychosocial HRQOL score. The findings are consistent with previous studies (31–33). Currently, several mechanisms are suggested explaining the associations between the family socioeconomic status and HRQOL of children (34–36). For example, Adler et al. (34) indicated that one pathway from family socioeconomic status to HRQOL is through exposure to different environments and adaptations to these environments. Children with low socioeconomic status may live in relatively poor neighborhoods, where they may be more likely to be victimized, and may experience peer aggression, and community violence (37). This may explain that they are more likely to have lower physical or psychosocial HRQOL. Another mechanism refers to the smaller access to material and social resources among children in families with a low socioeconomic status (36). Children from families with low socioeconomic status may have access to fewer social resources (i.e., services, goods, and social connections) than their counterparts, which may put them at risk for developmental problems, which may affect children's physical and psychosocial well-being (38). Therefore, the current findings support that health professionals should pay attention to children with low socioeconomic status backgrounds and good social support might help to improve these children's HRQOL.

Previous researchers have reported that, among children aged 9 and older, child with older age and living in a one-parent family were associated with lower psychosocial HRQOL (32, 39). Our study observed the same finding in a younger age group (6-year-old). However, our results showed that compared to girls, boys were more likely to have lower scores of psychosocial HRQOL. This finding is inconsistent with previous studies that reported lower HRQOL for girls, which was measured by KIDSCREEN (32, 39). Currently, gender differences in the HRQOL of children are even less clear, or are not found at all, more studies of this topic among younger children are needed in the future.

Regarding health-related lifestyle behaviors, previous researchers observed an association between health-related lifestyle behavior, such as physical activity (40) and screen time (41), and children's HRQOL. For example, Wu et al. (13) in a systematic review, reported that among generational population of children and adolescents aged 8 or older, a higher frequency of physical activity or less screen time, were both related to higher overall HRQOL. However, differently as expected, in the current study, no associations were observed between physical activity, screen time, and HRQOL. It could be possible that at the age of the children in this study (6-year-old), health behaviors do not yet show an association with HRQOL (42). The beneficial effects of physical activity or non-sedentary behavior on HRQOL might need to be assessed over a longer period of time (43).

This study found that health and well-being of the child and family (number of chronic conditions, emotional and behavior problems, and family environment) were negatively associated with children's physical and psychosocial HRQOL. Our results add to the evidence that chronic conditions in childhood may function as a stressor that affect the physical and psychological well-being of individuals (44). Thus, it is important for pediatric practitioners to pay attention to not only the development of the chronic conditions of the children, but also on their physical and psychological well-being.

Overall, socio-demographic characteristics, health-related lifestyle behaviors, and health and well-being of the child and family were studied in relation to both physical and psychosocial HRQOL. When studying the finding it appears that some factors might be specifically affecting HRQOL of early elementary school age children. For example, socioeconomic status was only associated with lower psychosocial HRQOL. Also, all indicators of health and well-being of the child and family were associated with both lower physical and psychosocial HRQOL. Therefore, priority might be given to children from a low socioeconomic status or with less favorable health conditions.

A major strength of this study is the large number of children being studied with detailed information on a broad range of potential determinants. However, several limitations should be considered. First, the cross-sectional of the present study did not allow to establish the causal relationship between all factors and HRQOL. Future work should examine the associations longitudinally. Second, child HRQOL was measured by the CHQ-PF28, which is a parent proxy measure. Thus, data presented in our study are from the parent's perspective. Nevertheless, previous work supports the validity of the CHQ-PF28 (26). We recommend future studies among children over 10 years to adopt the child self-report HRQOL questionnaire (CHQ-CF87). This could be used as an addition to the parent report HRQOL of the child. Taking both assessment would allow a comparison of parent perceived child HRQOL and HRQOL reported by the child him/herself. Third, the population in this study is on average 6 years old. Previous literature on determinants of HRQOL among younger children showed inconsistent results. Although the related data of current study was collected from 2008 to 2012, an ongoing birth cohort study offers good opportunity to generate an overall understanding of potential determinants of HRQOL in large general child population. Also, the factors included in this study cover all three domains. We recommend future studies to confirm our findings in more recent dataset.

## Conclusion

Our study investigated a wide range of factors covering three domains (socio-demographic characteristics, health-related lifestyle behaviors, and health and well-being of the child and family) related to child HRQOL. Public health initiatives to improve HRQOL of children should prioritize children from a low socioeconomic status or with less favorable health conditions from early age onward.

## Data Availability Statement

The datasets presented in this article are not readily available because the Generation R Study is subject to the Dutch law on medical research among humans (Wet Medisch-Wetenschappelijk Onderzoek met mensen, WMO). This law prescribes data analyses to be restricted to the topics defined in the information form and informed consent forms. It is not allowed by the WMO law that de-identified data are provided to others without restriction. The Medical Ethical Committee at Erasmus University Medical Center Rotterdam has approved the Generation R Study and supervises research leaders to conduct accordingly. Only after a written agreement about the use of the data made via the Technology Transfer Office of Erasmus MC, datasets can be requested by means of contacting the data managers (Claudia J. Kruithof, c.kruithof@erasmusmc.nl or datamanagementgenr@erasmusmc.nl) and the Director of the Generation R Study (Vincent Jaddoe, v.jaddoe@erasmusmc.nl). Requests to access the datasets should be directed to Claudia J. Kruithof, c.kruithof@erasmusmc.nl and Vincent Jaddoe, v.jaddoe@erasmusmc.nl.

## Ethics Statement

The study was approved by the Medical Ethics Committee of the Erasmus University Medical Centre (MEC 217.595/2002/202). Written informed consent to participate in this study was provided by the participants' legal guardian/next of kin.

## Author Contributions

YY, JY-H, AG, FE-L, and HR conceptualized and designed the study. YY performed the statistical analyses and drafted the manuscript. AG and HR supervised the data analyses. JY-H and AG contributed to methodology considerations. JY-H, AG, FE-L, and HR reviewed the manuscript for important intellectual content. All authors contributed to the article and approved the submitted version.

## Funding

FE-L received funding from the European Union's Horizon 2020 research and innovation programme under the Marie Sklodowska-Curie (Grant No. 707404).

## Conflict of Interest

The authors declare that the research was conducted in the absence of any commercial or financial relationships that could be construed as a potential conflict of interest.

## Publisher's Note

All claims expressed in this article are solely those of the authors and do not necessarily represent those of their affiliated organizations, or those of the publisher, the editors and the reviewers. Any product that may be evaluated in this article, or claim that may be made by its manufacturer, is not guaranteed or endorsed by the publisher.

## Appendix

**Appendix Table 1:** Univariate analysis of physical and psychosocial summary score between subgroups according to socio-demographic characteristics, health-related lifestyle behaviors and health and well-being of the child and family (*n* = 4,202).

	**Physical summary score (PhS)**			**Psychosocial summary score (PsS)**		
	**Mean ±SD**	***P*-value**	**Effect size**	**Mean ±SD**	***P*-value**	**Effect size**
**Child gender**		0.06	0.01		**0.038**	0.14
Girl	56.95 ± 6.34			53.26 ± 6.75		
Boy	57.01 ± 6.78			52.21 ± 7.35		
**Child ethnic background**		**<0.001**	0.24		0.745	0.04
Dutch	57.50 ± 6.14			52.72 ± 6.85		
Other western	57.25 ± 6.51			52.96 ± 7.27		
Non-western	55.75 ± 7.33			52.64 ± 7.50		
**Family situation**		**0.003**	0.12		**<0.001**	0.21
Two-parent family	57.13 ± 6.42			53.00 ± 6.81		
One-parent family	56.26 ± 7.24			51.27 ± 8.08		
**Maternal educational level**		**<0.001**	0.22		0.305	0.17
Low	55.84 ± 6.83			53.10 ± 7.42		
Mid	56.91 ± 6.92			52.53 ± 7.22		
High	57.37 ± 6.12			53.78 ± 6.79		
**Paternal educational level**		**<0.001**	0.23		**0.029**	0.10
Low	55.70 ± 7.63			52.28 ± 7.85		
Mid	57.20 ± 6.30			52.59 ± 6.88		
High	57.44 ± 6.07			53.06 ± 6.63		
**Maternal employment status**		**<0.001**	0.19		**<0.001**	0.02
Paid job	57.44 ± 6.09			52.89 ± 6.74		
No paid job	56.08 ± 7.22			52.77 ± 7.61		
**Paternal employment status**		0.110	0.13		0.066	0.06
Paid job	57.24 ± 6.28			52.87 ± 6.86		
No paid job	56.34 ± 6.69			52.44 ± 7.61		
**Net household income**		**<0.001**	0.21		**<0.001**	0.13
< €2,000/month	55.65 ± 7.42			52.14 ± 8.24		
€2,000–3,200/month	56.87 ± 6.79			52.20 ± 7.12		
More than €3,200/month	57.68 ± 5.91			53.22 ± 6.43		
**Physical activity**		**0.020**	0.10		**0.049**	0.10
<1 hour/day	56.68 ± 6.79			52.30 ± 7.17		
≥1 hour/day	57.35 ± 6.09			53.03 ± 6.81		
**Screen time**		**<0.001**	0.13		**0.005**	0.13
≥2 hour/day	56.35 ± 6.97			52.33 ± 7.32		
<2 hour/day	57.42 ± 6.22			53.03 ± 6.79		
**Number of chronic conditions**		**<0.001**	0.63		**<0.001**	0.38
0	58.70 ± 4.93			53.80 ± 6.70		
1	57.65 ± 5.81			52.92 ± 6.75		
2	56.34 ± 6.90			51.90 ± 7.30		
≥3	53.24 ± 8.61			50.98 ± 7.41		
**Emotional and behavioral problems**		**<0.001**	0.24		**<0.001**	1.17
Yes	55.01 ± 9.16			43.02 ± 8.90		
No	57.20 ± 6.25			53.47 ± 6.28		
**Family functioning**		**<0.001**	0.36		**<0.001**	0.45
Not pathological	57.28 ± 6.11			53.01 ± 6.79		
Pathological	53.63 ± 10.11			49.01 ± 8.98		

*The table is based on a non-imputed dataset. P-value are calculated by independent T tests or one-way ANOVA. Effect size calculated by dividing the difference in mean scores between subgroups by the largest standard deviation and interpreted as: 0.2 ≤ effect size ≤ 0.5 small difference, 0.5 ≤ effect size ≤ 0.8 moderate difference, and effect size ≥ 0.8 large difference. Bold values indicate statistical significance*.
